# The H1N1 pandemic: media frames, stigmatization and coping

**DOI:** 10.1186/1471-2458-13-1116

**Published:** 2013-12-03

**Authors:** Michael McCauley, Sara Minsky, Kasisomayajula Viswanath

**Affiliations:** 1 Department of Medicine, Medical College of Wisconsin, Milwaukee, WI 53226, USA; 2 Department of Social and Behavioral Sciences, Harvard School of Public Health and Center for Community-Based Research, Dana-Farber Cancer Institute, Boston, MA 02215, USA

**Keywords:** H1N1, Communication, Media frames, Latinos, Stigmatization, Stress and coping

## Abstract

**Background:**

Throughout history, people have soothed their fear of disease outbreaks by searching for someone to blame. Such was the case with the April 2009 H1N1 flu outbreak. Mexicans and other Latinos living in the US were quickly stigmatized by non-Latinos as carriers of the virus, partly because of news reports on the outbreak’s alleged origin in Mexican pig farms.

**Methods:**

In this exploratory study we examined the psychological processes of *cue convergence* and *associative priming,* through which many people likely conflated news of the H1N1 outbreak with pre-existing cognitive scripts that blamed Latino immigrants for a variety of social problems. We also used a transactional model of stress and coping to analyze the transcripts from five focus groups, in order to examine the ways in which a diverse collection of New England residents appraised the threat of H1N1, processed information about stereotypes and stigmas, and devised personal strategies to cope with these stressors.

**Results:**

Twelve themes emerged in the final wave of coding, with most of them appearing at distinctive points in the stress and coping trajectories of focus group participants. *Primary* and *secondary appraisals* were mostly stressful or negative, with participants born in the USA reporting more stressful responses than those who were not. Latino participants reported no stressful primary appraisals, but spoke much more often than Whites or Non-Hispanic Blacks about negative secondary appraisals. When interactions between participants dealt with stigmas regarding Latinos and H1N1, Latinos in our focus groups reported using far more *negative* coping strategies than Whites or Non-Hispanic Blacks. When discussions did not focus on stereotypes or stigmas, Latino participants spoke much more often about *positive* coping strategies compared to members of these same groups.

**Conclusions:**

Participants in all five focus groups went through a similar process of stress and coping in response to the threat of H1N1, though individual responses varied by race and ethnicity. Stigmatization has often been common during pandemics, and public health and emergency preparedness practitioners can help to mitigate its impacts by developing interventions to address the social stressors that occur during outbreaks in highly-localized geographic regions.

## Background

*I think that fear is not good for things. What fear does… is it causes one to make mistakes.*— Latino focus group participant

Infectious diseases have shaped the course of human history, resulting in more deaths than any other pathological cause [1]. And when diseases are thought to be lethal, people who perceive a great risk of infection sometimes cope with their fears by blaming new disease outbreaks on someone, or some group of people, who live outside of their own social sphere. In many societies, people whose national, ethnic or religious backgrounds differ from those of the majority group have historically been accused of spreading germs [2,3]. Such lay theories of disease transmission, often based on inaccurate risk perceptions and pre-existing social prejudices, serve two immediate functions: they temporarily soothe the anxiety felt by the accusers, while also stigmatizing those who are blamed [4,5]. This same sort of disease narrative captured the public imagination in April 2009 when A(H1N1), a novel strain of human influenza, appeared in Mexico and spread rapidly around the world. Some of the earliest cases were discovered near Mexican pig farms (hence, the “swine flu” nickname) and Latino^a^ immigrants from that country, and others, were often pronounced guilty by association. Soon, Mexican nationals and the products they produced were shunned across the globe; in the United States, some talk show hosts portrayed Mexican immigrants as disease vectors who threatened the health and security of other citizens [3,6]. This was especially unfortunate since disadvantaged groups in any society, including racial/ethnic minorities, suffer disproportionately during disease outbreaks [7,8]. US Latinos have high rates of chronic health conditions such as diabetes, obesity and asthma, which put them at greater risk for getting the flu – and for developing complications once they have it [9,10]. Compared to other social groups, Latinos have less access to healthcare, wait longer to seek medical help, and have fewer opportunities to obtain sick leave – or to utilize this benefit when they have it [11,12]. If the lessons of history hold true, the consequences of H1N1 stigmatization may deepen the sense of social marginality that many US Latinos already feel [13,14].

**Figure 1:**
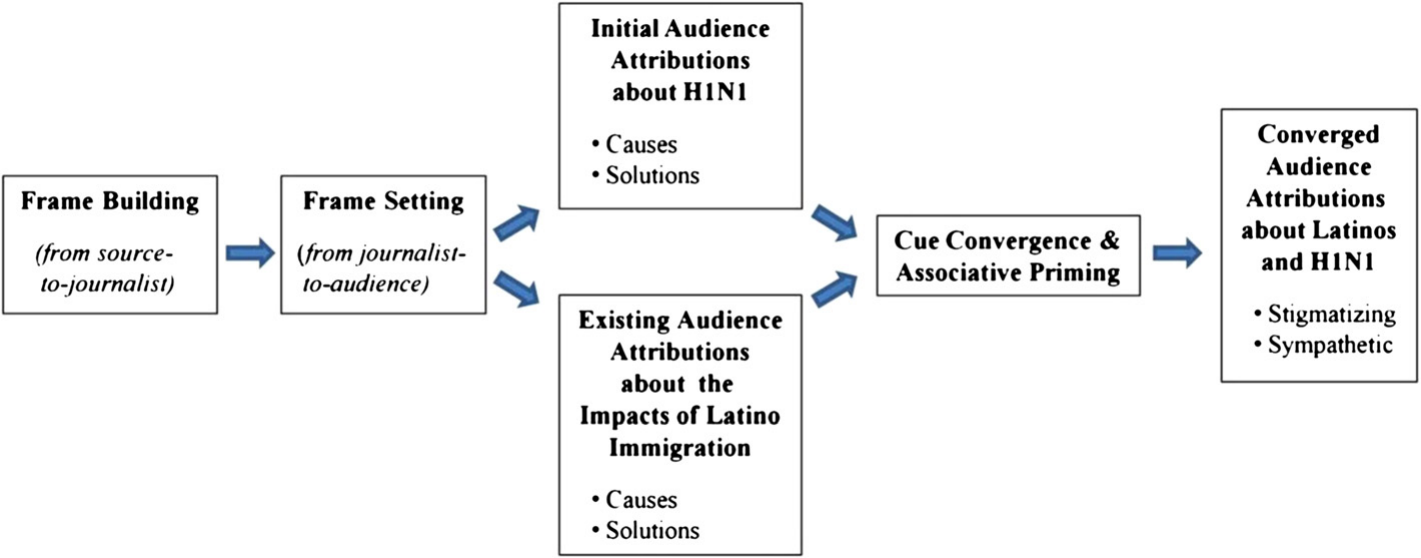
The role of framing and priming in the construction of media cues about H1N1 and Latinos.

In order to mitigate the negative consequences of stigmatization during pandemic outbreaks, public health officials must learn to recognize the dynamics that underlie this process, with special emphasis on protecting members of disadvantaged population groups [7,15]. Hence, it is important to understand how people’s fear of contagion during the 2009 H1N1 outbreak became associated with a broader set of fears about Latinos and the roles they play in American society. An emerging body of literature points to the competing goals of three interested stakeholder groups: media organizations and journalists; the public health officials that journalists relied upon for information about H1N1; and citizens who feared the spread of this disease and struggled to make sense of it. Figure 1 suggests one way in which competing frames about pandemic illness and US Latinos – including the social consequences of Latino immigration – collided and combined during the spring and summer of 2009.

Through the process of *frame-setting*, the news media actively develop the frames of reference that readers or viewers use to understand public events [16,17]. Yet the interpretations that journalists convey in their stories are often rooted in factors that lie beyond the simple reporting of facts. They are complex products of organizational constraints and professional judgments which are, in turn, influenced by the *frame-building* efforts of external sources [16,17] – including, in the case of H1N1, the US Centers for Disease Control and Prevention (CDC) and the World Health Organization (WHO). Among its many functions, WHO is responsible for monitoring disease outbreaks and warning the public when they cross borders and become international pandemics [18]. When cueing the CDC (and, hence, the US news media) about the threat of H1N1 in April 2009, WHO relied on a communication campaign that had actually been created to address a lethal outbreak of *avian influenza;* hence the ready analogies that some officials made to the deadly Spanish Flu pandemic of 1918 [19]. This strategy was a contributing factor in the sensational media coverage of H1N1 that developed in the US, a circumstance that challenged the ability of disease experts to communicate about the virus in a calm and dispassionate manner [20-22]. This campaign would ultimately cause a great deal of confusion, as the overall impact of the H1N1 in the US was significantly milder than public health officials first feared [23-26].

Nonetheless, nearly half of the respondents in an April 2009 national survey [27] were concerned that they or someone in their family might get sick. 59 percent said they had begun to wash hands or use hand sanitizer more frequently, and 25 percent said they were avoiding malls, sporting events or public transportation. And since media reports pegged the origin of H1N1 to Mexican pig farms, heavy coverage of the outbreak likely helped to activate a broader set of fears which held that Mexicans and other Latinos *might actually be spreading the disease.* Indeed, 20 percent of respondents to the same survey said they would avoid people who they *thought* may have recently travelled to Mexico; another 17 percent said they would avoid Mexican restaurants and stores [27].

In exploring these processes, we do not suggest that certain groups of fearful US citizens made conscious decisions to vent their frustrations on Latinos. Instead we posit a subtler form of bias that medical historian Howard Markel describes as a “fear of people we do not know or who look different”. In an interview with *msnbc.com*, Markel – a senior CDC consultant on pandemic preparedness – said that when “you take the fear of the unknown that already exists and then combine that with a real or perceived threat that is contagious disease… it’s explosive” [3]. Following this train of logic, we can begin to understand how the fear of contagion can become associated in an anxious person’s mind with a generalized fear of minorities [28]. Social psychologists suggest that this sort of associational trajectory develops in a part of the human brain that stores the network of interconnected cognitions which comprise long-term memory [29]. Given a vast array of possible connections, any singular concept that is encoded into memory (e.g.*,* anxiety about flu outbreaks) may become associated with other constructs (e.g., the urge to assign causes and cures). And every time these concepts are activated in tandem, the connections between each member of the “concept set” become stronger. Regarding our study, the presence of threatening linguistic “cues” in news stories about pandemic illness may trigger or activate other constructs that are stored in long term memory (e.g.*,* fears that immigrants are disease vectors). This process of *cue convergence,* and the resulting activation of associated constructs (*associative priming*) [29], represents one pathway through which a person’s memories about flu pandemics may combine with historical or latent racial/ethnic fears to make incidental contact with Latinos seem like a dangerous proposition (Figure 1).

To be clear, these processes do not imply that the news media are solely or even mostly to blame, as high-profile talk show hosts (who do not follow the professional conventions of journalists) arguably took a more active role in developing and propagating certain lay theories about the pandemic. Some dubbed it the “fajita flu” and took the growing number of cases as evidence that the uncontrolled flow of illegal aliens across the southern US border posed a clear and present danger; others suggested that the H1N1 outbreak could be part of a larger conspiracy in which terrorists targeting the US would infect Mexican immigrants and turn them into walking, talking weapons of germ warfare [3,5,6,30]. Given the public’s fear of the unknown, and the ways in which the media stoked this fear with a variety of associative cues, many US Latinos were likely *stigmatized* and *stereotyped* during the pandemic. In general terms, stereotypes are cognitive shortcuts that help a person to develop quick, superficial “readings” of people from other social groups [15]. Whether conscious or implicit, stereotypes are a frequent by-product of stigmatization – the “marking” of certain individuals or groups according to a socially-conferred judgment that they are somehow tainted, or less than, members of the majority population group [31]. Stigmatization stems from the fears that people often face during times of uncertainty, including public health emergencies like the H1N1 pandemic [30-33]. Apart from disparaging media portrayals of Latinos, a series of interviews with US public health advocates in 2009 showed that Latino seasonal farm workers often felt stigmatized or shunned by other people in the communities where they lived or worked [5]. Government officials, Latino advocacy organizations and public health groups moved to denounce these occurrences [34,35], yet it is likely that stigmatization exacted a toll. People who are subjected to social avoidance or rejection often internalize the stigma they experience, a process that leads to heightened psychological stress and anxiety – and, in some cases, to an increased susceptibility to illness [15,36,37]. It is also important to note that the process of stigmatization may negatively impact the psychological and physiological health of people who *hold* derogatory or prejudicial beliefs about members of another social group [38-40].

**Figure 2:**
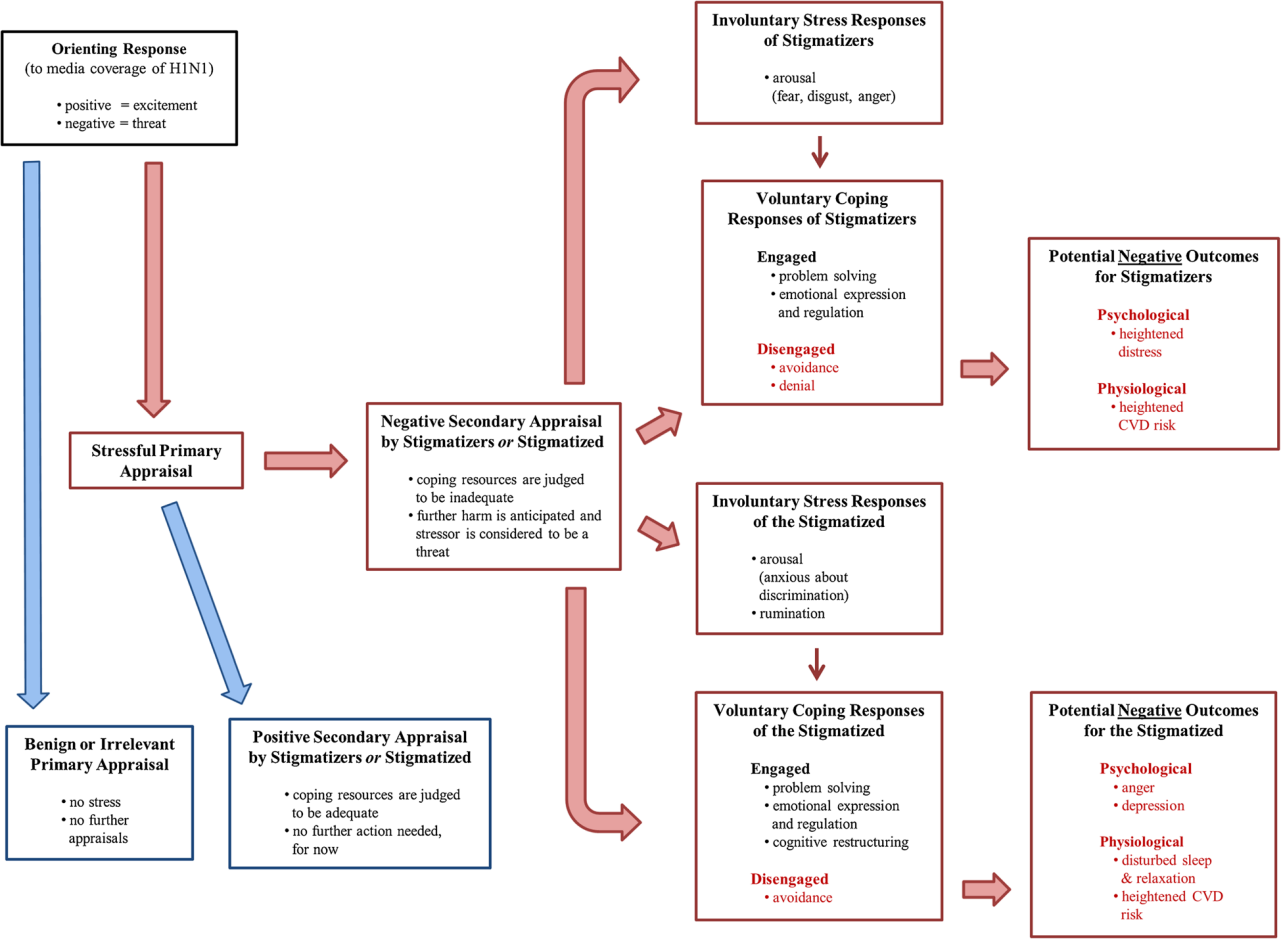
A stress and coping framework for the processing of H1N1-related media discourse.

How did US residents deal with the stressful circumstances of the 2009 pandemic? One plausible framework for understanding this is Lazarus and Folkman’s transactional model of stress and coping [41]. We have adapted this model (Figure 2) in a way that describes a process that both stigmatizers and the stigmatized go through when confronted with distressing social situations.

In this model, stressful experiences are conceptualized as person-environment transactions which depend, first of all, on the impact of an external stressor (e.g.*,* media coverage of H1N1). Research suggests that we humans are “spring-loaded” to evaluate our informational environment, and that our initial evaluation of stressors may happen subcortically, prior to any conscious awareness or emotion [42,43]. This *orienting response* can be sudden and unintentional; and since it functions as a psychological early warning system, it often assigns priority to stimuli that are threatening in nature [43]. From the standpoint of those who stigmatize, it is thus possible that certain media cues about pandemics can quickly activate elements of historical or latent stereotypes about Latinos that are stored in long-term memory – without any sort of conscious intent or present-day belief on the part of a person having this response [44,45]. These same media cues may activate a different sense of apprehension on the part of Latinos who have often, and in various ways, felt the burden of prejudice and discrimination during times of widespread social unease, including the early stages of pandemics.

The next step in our model is *primary appraisal,* in which individuals evaluate the demands of the potential stressor to determine whether it is important to their own well-being or the welfare of people they hold dear. When people judge the stimulus at hand to be irrelevant or benign, they spare themselves from psychological stress [41,42,45]. But when a threatening event seems likely to produce negative consequences, their agitated response hastens a *secondary appraisal.* Here, people evaluate the resources they might use to mitigate the threat. During this process, individuals may evaluate whether their available *psychological* resources (e.g.*,* self-esteem, optimism, self-control), *personal* resources (e.g.*,* income, education, occupational status) and *social* resources (e.g.*,* family resilience, social support, ingroup identification and evaluation) will help them to stay resilient in times of danger [33,46]. During secondary appraisal people ask, in effect, whether they have what it takes to manage the challenges of a threatening circumstance [41-43,45-47] (Figure 2).

When individuals go through primary and secondary appraisal, yet continue to experience the stressor in a threatening manner (e.g.*,* because of a steady stream of H1N1 news) they experience psychological stress and must find ways to respond. Our model of stress and coping acknowledges the distinction between *involuntary responses to stress* and *voluntary coping responses.* Regarding the first category, people may have involuntary emotional, behavioral, psychological and cognitive responses to stress that do not serve to regulate stressful experiences, including physiological and emotional arousal, rumination and intrusive thoughts, and impulsive actions. Whether conscious or unconscious, these responses “are experienced as being largely outside of the person’s control” [48]. Coping, on the other hand, denotes “conscious volitional efforts to regulate emotion, thought, behavior, physiology and the environment in response to stressful events or circumstances” [48,49].

If the resources a person can draw upon seem sufficient for dealing with a stressor, that person may select a *positive-engaged* coping strategy^b^ such as problem-solving, finding a good balance between emotional expression and emotional regulation, or cognitive restructuring – a strategy discussed more thoroughly in our Results section. To give an example of a positive-engaged problem-solving strategy, a White person who is troubled by the prospect of Mexico’s role in the H1N1 outbreak – but confident in his/her ability to cope – may reduce stress by learning all that he/she can about the virus through respectful conversations with Latino acquaintances. But if news coverage of the outbreak stimulates a threatening response, and the individual does not feel up to the challenge, that person may choose a *negative-disengaged* coping strategy such as avoiding or denying the problem, or antagonizing people who seem threatening (e.g.*,* Latino restaurateurs or travellers who have just returned from Mexico). Members of traditionally stigmatized groups may also employ the whole range of voluntary and involuntary responses to stress. Of special concern to public health practitioners, people who feel stigmatized during pandemic outbreaks may turn inward or isolate themselves from social contact, making it more difficult for them to receive appropriate medical care [11,12]. Finally, we suggest – by inference, not empirical evidence – that the strategy a person chooses for coping with a public health crisis will ultimately have an impact on disease susceptibility and health outcomes (Figure 2).

In this study, we will demonstrate the ways in which public health officials can recognize and understand the potentially harmful dynamics that underlie the stigmatization process during pandemic outbreaks. Specifically, we will utilize the model of stress and coping outlined above to inform an exploratory analysis of five focus group sessions conducted in New England during the H1N1 outbreak of 2009. By revisiting these transcripts with an eye toward the structural elements of our model, we will highlight the primary and secondary appraisals of focus group participants following their exposure to media reports about the pandemic. We will show, in various ways, how the appraisals these people made are linked to the involuntary and voluntary responses that resulted. And we will show how recent research on the psychological makeup of stigmatizers and the stigmatized can offer guidance on the design of anti-stigma interventions whose effects may be longer-lasting than traditional education-based interventions. This study makes no claim to generalizability in the broadest social-scientific sense. Instead, we follow Sim’s conception of *theoretical generalizability*[50], with the intention that our results will produce theoretically useful insights for others who may conduct future studies about stress and coping during public health emergencies.

## Methods

### Participants and setting

Our focus groups were originally conducted as formative research in support of a larger study of public health emergency preparedness (PHEP) communications. This project, which included a public opinion survey, sought to better understand the information sources people used when developing emergency preparedness plans, the level of trust they had for various information sources, and the knowledge and perceptions they held with respect to the 2009 H1N1 outbreak.^c^ Participants were recruited through newspaper advertisements and flyers, and from the rosters of other projects undertaken by a major research institution in New England. The goal for each focus group was to recruit 8-10 participants, ages 25 and older, from diverse ethnic, racial and socioeconomic position (SEP) groups in the New England locations described in Table 1. A total of 46 participants were recruited for five focus groups (two in City A), with an even representation of both rural and urban residents.

**Table 1:** Demographic characteristics of cities/towns where focus groups were held^*^

	City A^**^	**City B**	**City C**	**City D**
**Population**	600, 980	7,380	72,043	7,827
**Race/Ethnicity**				
White	56%	85%	49%	98%
Black	24%	11%	5%	0.4%
Hispanic	16%	7%	60%	1.2%
**Education**				
≥ HS	84%	82%	58%	84%
≥ College	27%	31%	10%	17%
**Income**				
Median HH Income	$48,729	$60,752	$27,983	$49,310
Families < Poverty Level	17%	4%	21%	4.5%
Individuals < Poverty Level	21%	4%	24%	6.8%

^*^Figures are based on 2000 and 2005-2007 US Census data.

^**^Two of the five focus groups were held in City A.

A large proportion of participants came from low-SEP and underserved population groups (See Table 2) and a Spanish-language focus group was conducted in City C, where the Latino population comprised nearly 60 percent of the city’s overall population. (Table 1) Participants ranged from 26 to 72 years of age and had educational levels from 4th grade to Bachelor’s degree (Table 2).

**Table 2:** Participant characteristics, by focus groups

	City A^*^	**City B**	**City C**	**City D**	**Total**
**# of participants**	16	9	10	11	46
**Gender**					
Female	9	6	6	8	63%
Male	7	3	4	3	37%
**Age**					
Range	27-61 yrs	32-70 yrs	26-65 yrs	47-72 yrs	26-72 yrs
Mean	45 yrs	53 yrs	45 yrs	61 yrs	51 yrs
**Race/Ethnicity**					
White	1	8	x	10	41%
Black	13	x	x	x	28%
Hispanic	2	1	10	x	28%
Other	x	x	x	1	3%
**Education**					
< HS	7	x	x	x	15%
HS	7	1	8	5	50%
≥ College	1	8	2	6	35%
**Income**					
HH < poverty level	8	1	2	5	35%

^*^Two of the five focus groups were held in City A.

### Focus groups

The focus groups, approximately 90 minutes in length, were held at locations that were easily accessible to participants. Following standard protocol, participants were welcomed to the discussion by investigators, informed about the purpose of the research and given a set of ground rules, including the expectation that they respect and listen to each other. Following a briefing on recording procedures, each focus group was audio-taped and transcribed. Most of our focus groups (Cities A, B & D) were conducted in English by a cultural anthropologist with extensive experience in facilitating focus groups. The group in City C was conducted by a Spanish-speaking moderator who also has extensive experience in focus group research. (See Additional file 1 for a copy of our focus group script).

### Analysis

Transcripts were analyzed according to a standard comprehensive qualitative analysis method, which involved a three stage coding process. In the *structural coding* phase, the principal analyst coded textual elements in each transcript that corresponded with the ways in which our respondents first encountered H1N1-related stressors, appraised the attendant level of threat, and developed strategies to try and mitigate the threat. This portion of the analysis was enhanced by the use of *NVivo* (QSR International), a state-of-the-art ethnographic data management software program. The second wave of analysis followed the *immersion/crystallization* method [51], a process that involves immersing deeply in key portions of the coded data – then backing away from it at regular intervals for the purpose of reflection and second-level theme formation. When this wave of coding was complete, revised output reports from *NVivo* were generated and scrutinized. All authors reviewed the results of these coding processes and contributed to the comprehensive summary of qualitative findings detailed in the next section.

### Ethics and consent

The Institutional Review Board at the Harvard School of Public Health determined that the focus groups conducted for the present study were exempt from any regulations pertaining to the conduct of research on human subjects. All focus group participants completed appropriate consent forms and each was paid an incentive for his or her participation.

## Results

Twelve themes emerged in the final wave of coding, with most of them appearing at distinctive points in the stress and coping trajectories of our focus group participants (Table 3).

**Table 3:** Summary of study themes

	**Themes**	**Examples**
**Primary appraisals**		
**Stressful**	What are we in for?	“I don’t know what I would do if anyone in my family got sick”
		“Even the young and normally healthy people are getting sick”
**Secondary appraisals**		
**Positive** (“meet the challenge”)	Plans and Precautions	“Taking precautions against viruses… is just part of my routine”
**Neutral**	It’s not so bad	“I never get the flu, so I really wasn’t concerned”
**Negative** (“threatening”)	Nobody Knows”	“I don’t think anyone has a full understanding of this flu”
	It’s In The Air	“H1N1 is in the air. If you’re exposed to it, you will get it”
	Isolation	“We’re isolated in my neighborhood/We don’t know, or help, each other”
**Coping strategies**		
**No Stereotype Discussed**		
Positive-engaged	Too Much News, Too Many Germs	“Now, I’m more alert as to how you can catch germs”
Negative-Passive	Who Can I Trust?	“I think the government is just tryin’ to get people worked up”
**Stereotype Discussed**		
Positive-engaged	Protection, not Isolation	“Take precautions, but live your life… and care for people in need”
	Raising Consciousness	“Widespread fear has economic consequences - especially for Latinos”
Negative-Passive	Subtle Stigma	“Is our lifestyle ’cleaner’ than the lifestyle in Mexico?”
	We’ve Heard This Story Before	“Americans are not very sensitive to minorities during disease outbreaks”

### Orienting responses and primary appraisals

#### “What are we in for?”

The initial orienting responses that people have to a stimulus are “rapid and automatic” – quick reflexes that “assign an initial processing priority through the allocation of attentional resources” [43]. Our focus group data do not contain direct evidence of this cognitive early warning system. But they do contain evidence of primary appraisals, the initial analyses in which people decide whether the stimulus at hand (i.e.*,* news about the flu outbreak) is threatening to themselves or someone they care for. In our study, most of the primary appraisals we could discern were stressful in nature. Take the comments of these two participants, for example – first a White woman from a rural community, then a Black woman from a major city.

I remember the first outbreak of the swine flu, and I’m like, “Oh, crap!” When they’re talking about it crossing the borders and all of that… I mean I, too, am very concerned.

You know, I, I just panicked, you know, because I don’t know what I would do if… you know, anybody in my family was sick with the swine flu.

Reactions like these are hardly surprising, as primary appraisals are often based on information that is seemingly dangerous – and incomplete. And since these appraisals are often suffused with negative emotion, it is also no surprise that our participants would focus on news about severe cases and rapid propagation.^d^

**F:***‘Cause usually when you get an outbreak or something like the flu, or something that kills people, they always say it’s little babies or older people… But then we’re seeing like healthy people, you know, people who are in, um…*

**M:***…in the best of shape.*

**F:***Yeah, and… who were, you know, younger. And it was still… they were still gettin’ very sick from it. So that was kind of scary, because it was, like, across the board.*

### Secondary appraisals

#### “Plans and precautions”

Secondary appraisal occurs when anxious individuals evaluate the availability and effectiveness of the resources they can muster in dealing with the perceived threat. Some participants from City B, which had the highest mean levels of education and income in our sample, felt reasonably safe since health officials, public safety officers and other civil servants had already made effective public communications about their plans for containing a flu outbreak. At least some participants in all of our study locations felt they could stay reasonably safe by simply following the precautions mentioned by journalists, healthcare providers and other sources.

One woman from City A, a large urban area, noted that her occupation prepared her quite well for the task of warding off viruses.

And generally, it’s part of my routine, because I work with kids. So, everything that I touch… as soon as I move, I gotta’ wash it. So it’s like, when that outbreak came out? It was like nothing new. We were always doing that.

#### “It’s not so bad”

Some participants made neutral appraisals of H1N1, taking note of the pandemic but concluding that no action was necessary. Three people from City A reasoned that while other people were vulnerable to H1N1, they would probably be OK. And a woman from City D, a small, rural community, was among several participants who expressed only minimal concern, saying the outbreak was not as bad as other people might think.

Since I never have the flu, you know, I really wasn’t concerned. Then I listened a little more… ‘cause I figured all the amount of people that have had it, and the few people that have died from it… it’s all relative. And I’m really not that concerned.

#### “Nobody knows”

However, most people in our focus groups made negative secondary appraisals, judging that their coping resources might not be enough to keep them healthy. In City A, where income and education levels are comparatively low, participants in one focus group had trouble comprehending all the fast-breaking details about H1N1. Some members of this group blamed the situation on health experts and news reporters who failed, in their view, to provide enough useful information.

**F1:***I don’t think we got the full understandin’ of the swine flu. And people can’t get over it.*

**F2:***I don’t think they have a vaccine for it.*

**M1:***I don’t think they have enough.*

**F3:***Yeah, they prompt us sayin’ that they did. But they… they really don’t. They haven’t figured it out how to make it yet.*

And a man from the other focus group in City A said the flu outbreak shook his sense of confidence that biomedical scientists even know how to protect the public from harm.

You know, we have kind of the top scientists and everything in the world… It amazes me that we can do so much for other countries and come up with so many different things for them. But somethin’ hit at home, we are completely baffled and lost with it.

#### “It’s in the air”

Some focus group participants from City C – all Latinos of Dominican descent – also felt the H1N1 outbreak might overwhelm their ability to cope. On the whole, people in this group were quite knowledgeable about the ways in which flu is transmitted, noting that viruses are simply “in the air” and that there is little that a person can do to hide from them.

#### “Isolation”

Even more important, people from City C were afraid that in responding to the threat of H1N1, healthcare workers and government officials would actually deepen the sense of physical and social isolation they already felt. As members of an immigrant culture that is defined, in part, by the fear of arbitrary deportation, these people were naturally wary about the impacts of medical quarantine. In particular, some of the women in this group feared the disease could wreak havoc in their tightly-knit community – if health officials required, for example, that they care for infected children at home.

And some older members of this group were painfully aware that *social support –* a key factor in resilient responses to pandemic disease – was not available to them in the United States in the same way they encountered it back home.

**M:***If a method was available – a neighborhood, a club where people could get reunited, and get to know each other better, and talk – then you would know the people in your neighborhood… share together, you understand me? But each person lives isolated one from the other in their rooms. You enter your house and lock yourself, and no one knows each other most of the time.*

**F:***Because we are afraid of each other.*

**M:***That is what is happening, that sometimes there isn’t friendship. There are no friends or anything because we don’t believe each other.*

### Coping strategies and other responses to stress

#### “Too much news… too many germs”

When people continue to perceive environmental stressors as threatening despite their available stores of mitigating resources, they search for ways to respond – to manage the negative emotions they experience or, perhaps, to alter the stressful circumstances that cause these emotions in the first place. Some focus group respondents seemed unable to find enough resources to calmly address the challenges of H1N1, and coped by simply avoiding news coverage of the pandemic. And some coped with the glut of H1N1 news in ways that suggested a pre-existing set of compulsive behaviors designed to keep themselves, and their families, germ free – perhaps, as a means of diverting their attention from the constant barrage of H1N1 news. For example, a group of women in City D developed an impromptu checklist of items that should always be cleansed with antibacterial lotion or wipes, including telephones, shopping carts, money, pens used in public – and, in some cases, visitors to one’s home.

#### “Who can I trust?”

Some anxious respondents became overwhelmed by the sheer amount of information available about H1N1, and by the fact that news coverage of the outbreak contained a fair amount of conflicting information. In some cases people seemingly drifted into a state of denial about the significance of an H1N1 pandemic. According to this elderly White woman from City D, too many people were making a fuss about the pandemic – “saying this and that” until it all seemed like so much noise.

And I think the… the government is puttin’… puttin’ things into peoples’ mind… [Along] with a lot of other things, I think the government is just tryin’ to get people worked up.

An African-American woman from City A, who listened more carefully to the news, complained about mixed messages from the highest echelon of federal government.

Other people were sayin’, “Oh, it’s safe. It’s … I’m not gonna’ worry about it. And then Joe Biden comes on and he’s sayin’ “I’m not gettin’ on a… if it was my family member, I’m not… I wouldn’t put ‘em on a plane, or train or anything.” So then you’re like, “Well, who am I supposed to believe?”

At one level, these comments provide evidence of an involuntary sort of rumination about the ultimate meaning of conflicting news reports. At another level, they suggest a fraying of trust for some focus group participants regarding the ability of government officials to adequately protect US residents during pandemic flu outbreaks – or, at very least, to communicate with them in a manner that is clear, concise, and sufficiently useful.

#### “Subtle stigma”

Many people in our groups were skeptical about traveling to Mexico anytime soon. In most cases they simply shared an understandable impulse to stay away from the epicenter of a threatening disease. But the tone of the discussion shifted, at times, in ways that suggested an aversive response – a subtle form of stereotyping with respect to Latinos, including the propagation of lay theories about differing lifestyles in the US and Mexico. One interesting thread developed in the focus group that met in City B (small town, high SES, mostly White) when an anxious woman grilled another participant – a local health official – about the severity of the 2009 pandemic.

**F:***The mass amounts of people dying. It’s, it goes back to my whole thing of… why is it that this country [USA] didn’t have the amount of deaths that other countries did? Are we doing something different? Are we more, um… do we have better resources through the CDC or something…*

**M:***We have… we have better resources.*

**F:***…to do that? But… how could we use that to be able to, to… to keep that from being as bad as,* per se*, Mexico the NEXT time it comes around… if it does?*

At another point in the discussion, this same woman confessed a sudden aversion to continuing with routine family outings at restaurants and other public facilities in a large city, located about an hour’s drive away.

I kind of looked at it and said, “Well you know somethin’? That, that little evening out doesn’t have to happen this month or next month. We can push it off and wait… till later.”

Taken as a whole, these comments provide evidence of the speaker’s desire to avoid uncomfortable feelings about the threat of contamination – first by favorably comparing her own social reference group (i.e.*,* “White America”) to that of people who live near Mexican pig farms, and then by physically avoiding places she deemed to be risky. The lone Latino participant in City B listened attentively to these comments and offered a bit of “push-back” regarding the economic impact of such behavior on restaurateurs and other Latino businessmen.

#### “We’ve heard this story before”

When studying stigmatization and other impacts of social stratification, it is important to understand the ways in which historical context may shape a person’s views of present-day experiences [52]. For instance, several Latino participants in City C spun a fascinating thread of discussion when asked about their information-seeking habits during the 2009 pandemic; indeed it was the first of two instances in which Latinos showed evidence of *cognitive restructuring*[48,49]. In the example that follows, this sort of restructuring helped people to redefine an unpleasant situation in a culturally-relevant manner that, in the end, also helped to mitigate stress. One man said he had learned on the Internet of a 1982 outbreak of *African swine fever* (ASF) in his native land. ASF is extremely lethal for pigs and US agricultural officials – fearful the disease would spread, and then decimate the US pork industry – worked with Dominican and Haitian officials to exterminate hundreds of thousands of infected native pigs [53,54]. This fact did not escape the attention of other focus group members.

**M1:***All of the pigs. It finished off everything, and they had to bring in a new race. And that is how all of the pigs, the tumaron and the black one…*

**F1:***And the black one.*

**M1:***It finished everything and a new type of white pig came in.*

**F1:***Americans.*

**F2:***Another race.*

**M1:***American ones, another race. So say… well, if this time we can’t finish all of the people…* [Everyone laughs]

At this point, a female participant said her mother, formerly a pig farmer, believed the “swine flu” of 2009 was not new – that it was, in fact, the very same disease that ravaged native Dominican pigs in the late 1970s and early 1980s. Though this argument is literally untrue, it offers an important fulcrum for analysis. ASF is *not* the same as H1N1, most notably because the ASF virus has no direct impact on humans [54]. Still, it is undeniable that some Dominican and Haitian pig farmers lost a significant portion of their livelihood because of a disease outbreak in which US officials helped to kill thousands of native pigs. Thus, focus group participants in City C used memories of the ASF outbreak as a cognitive shortcut for understanding the social hazards *they might face* during an H1N1 outbreak in the United States. By intimating that “we (*Latinos*) have seen this (*American*) flu before”, they were able to bring a sense of shared meaning to the task of coping with a threatening circumstance whose solution seemed to lie beyond their control.

#### “Protect yourself but use common sense”

To be sure, our focus group participants – especially those who lived in low SEP, urban settings – also engaged in positive coping strategies, using a blend of problem-solving and appropriate emotional expression. Several people showed a deep empathic streak upon hearing initial reports of the flu pandemic, including an African-American man from City A who wondered if international health agencies would be able to supply Mexican citizens with face masks that did a better job of filtering viruses. Closer to home, some participants noted awkward moments at church, where nervous preachers advised against friendly contact – and were promptly ignored. Latinos from City C spoke about their efforts to balance flu prevention measures with the practical demands of social life. And two women from City A launched a spirited bit of push-back against the prevailing US culture of germ phobia, noting that children who grow up “playing in the dirt” have healthier immune systems than those who are raised in a sanitized “bubble”.

#### “Love God, then call 911”

One brief, but fascinating exchange provides a glimpse of a potentially resilient strategy, a bit of cognitive restructuring that Latinos in City C apparently use when coping with public health emergencies. It began when the moderator asked “How are you prepared?”

**M1***: Well because I always think that there is a special protection over me that comes from [God] the Father.*

**F1:***That’s true.*

**F2:***Amen.*

**M1**: *I’ll always feel safe. Yes.*

**Moderator:***And the rest of you? Do you know what to do in case of an emergency?*

**F1:***Love God.*

**F2:***Love God and then call 911.*

**F1:***God can make a miracle right there.*

This passage offers a glimpse of a Latino-American coping style that Florez *et al*[55] refer to as *destino –* a deep sense of conviction that one can overcome any obstacle in life by combining the grace of God’s blessings with a healthy dose of self-efficacy and hard work. The *destino* narrative, which surfaced frequently in the City C focus group, stands in contrast to the commonly-held view that US Latinos approach a variety of intractable health problems with a fatalistic attitude. Latinos in our focus groups showed no signs that they would “give up” when faced with the challenges of H1N1, or with the attendant difficulties of stigmatization. Armed with the belief that “God helps those who help themselves”, they spoke of many ways in which their abiding faith in a higher power helped them to meet and surmount many of life’s challenges. In this manner, they were able to reduce the impact of the emotional arousal that followed heavy media coverage of the flu pandemic.

#### “Raising consciousness”

Finally, several participants responded positively, and adaptively, in discussions that focused directly on the flu-related discrimination that Latinos sometimes faced in 2009. The best example comes from City B, where the “anxious participant” described above joined a highly charged interaction between two other members – and got more than she bargained for in return. It began with an observation by the lone Latino member of this otherwise White group that the media were unjustly hatching “theories” that blamed the spread of H1N1 on people born south of the US border.

**M1:***Blame it… blame it on Mexico, not us.*

**F1:***Right. That’s right. And I think that’s…*

**F2: *We’re blamed enough. We don’t need any more!*** (Chuckles)

**F1:***With the number of people that, that I had a conversation with, you know, the general thought was: “Oh, yeah, it came from Mexico, and they just don’t have the regulations like we have on meat and food.” And, you know, “We’re on top of things here in the United States. It can’t possibly touch us.” And that’s where the disillusioning piece comes into play. And if that helps you to sleep at night… well, I don’t know. I’m not so sure that that’s a good, right way to handle that.*

In effect, the defensive comments of the anxious woman – which appear above in bold-faced type – triggered a quick response by another White woman in the group, who spoke about the unforeseen consequences H1N1 stigmatization in a manner that was emotionally balanced and appropriate. Later, the Latino businessman who started this conversation highlighted the possible impacts of subtle or implicit stereotyping.

I mean, right here we have an example of [a restaurateur who] lost revenue because a trip that [F2] normally takes into [City A] to do something, she chose not to do. And, and that’s happening… because people are concerned about what happens at the restaurant. Yeah, we’re talking health here… but definitely, an impact on the economy.

These exchanges in City B are significant because of the unexpected opportunity they offered participants to actively confront and contest certain implicit racial/ethnic biases that link Latinos to the spread of H1N1. This narrative took shape based on the comments of the group’s only Latino member, a well-educated and well-traveled businessman. Safe within the confines of the focus group setting, he had an opportunity to voice his concerns calmly and effectively – a discursive avenue that is often unavailable to members of racial/ethnic minority groups when discussing these matters in public.

## Discussion

Our study has attempted to explain the ways in which two separate strands of concern about disease, and the “exotic others” who threaten to spread it, came together in the spring of 2009 to make it seem as though Mexican and other Latino immigrants were largely responsible for the spread of H1N1 onto US soil. It has also attempted to explain the ways in which some US residents either created, or coped with, the stigmas and stereotypes that soon became evident in this charged emotional environment. Our focus group data do not contain direct evidence of the involuntary stress responses that developed when participants first encountered news reports about H1N1. But comments about primary appraisals – their initial assessments about whether H1N1 would pose a threat to themselves or to loved ones – suggest that most participants did become emotionally aroused by H1N1 news. There were subtle differences in the precise kinds of stress these people mentioned, depending on their race and ethnicity. For instance, we found little evidence that Latino participants first considered the spread of H1N1 to be a significant threat to themselves or their loved ones. It is possible that respondents from City C found it difficult to discern such a threat, given the many stressors that people in their community face on a daily basis. Some Whites, on the other hand, became aroused by H1N1 news in a manner that suggested fear, disgust and anger. In terms of secondary appraisals, our focus group participants reported having more negative experiences than positive ones – and this trend was most pronounced among Latinos. While they did not perceive an immediate or outsized threat from the flu, these participants did question whether they had enough resources to combat it – noting the ubiquity of airborne viruses and the lack of social support mechanisms in the event that one actually becomes ill.

In terms of voluntary coping strategies, both Whites and Hispanics addressed their anxiety over H1N1 news, at times, through affirmative problem-solving strategies. People from both groups also made conscious efforts to balance their desire to express feelings about H1N1 with a perceived need to do so in an even-handed way. When focus group discussions *did not* focus on flu-related stereotypes or stigmas, Latino participants reported much greater use of positive-engaged coping strategies than Whites or Non-Hispanic Blacks. When stereotypes *were* part of the conversation, Latinos reported fewer positive strategies and many more negative-disengaged strategies. This observation can be interpreted, perhaps, by way of reference to David Williams’ assertion that we humans “tend to think with our hearts” – that the emotional context which surrounds sensitive public conversations has a powerful impact on whether participants feel empathy or antipathy toward people from other social groups.^e^ Interestingly, Hispanic participants engaged in cognitive restructuring in order to make sense of H1N1 (and related stigma) within their own cultural framework. This allowed them to understand the prospect of present-day discrimination in light of their perceptions of historical discrimination at the hands of Whites. This coping strategy also became manifest when Latinos fused their sense of natural self-efficacy with strong religious beliefs, embodying the culturally-specific trait known as *destino.*

While our results cannot be broadly generalized, they do contribute to the scholarly literature in at least three important ways. First, our work corroborates other studies that describe the racial and ethnic tensions that sometimes lurk beneath the surface of public discourse on H1N1. Second, we demonstrate that two strands of social-psychological theory, cue convergence/associative priming and a transactional model of stress and coping, can help public health practitioners understand more clearly that disease outbreaks are *social stressors –* threatening stimuli that can activate a set of latent fears some people may hold about members of other racial/ethnic groups. Finally, our work has implications for public health and emergency management officials who want to mitigate the impact of stigmatization during future outbreaks. It is important to note that anti-stigma interventions are not likely to curb this problem completely. Since subgroup identification is an important basis for sifting one’s way through a sea of complex interpersonal interactions, it is likely that these interactions will always embody some sense “us” and “them” [31]. Despite this cautionary note, scholars of stigma – and of H1N1 stigma, in particular – do suggest certain fruitful possibilities for intervention. First, they maintain that local officials should make people in their communities aware of the prospects of stigmatization *before* the next pandemic hits. For example, they can teach their staff members about the harm that results from stigmatization; work with the media to tell their story in a way that does not single-out one group or location as the “source” of disease; create mechanisms for the rapid portrayal of accurate information on disease risk; and ensure that people who feel stigmatized have a means of expressing their concerns [15]. Second, other community and faith-based organizations should be reminded to transmit accurate messages that address people’s concerns about getting sick; to actively work to mitigate harmful rumors and misinformation; and to model respectful and compassionate behavior when interacting with members of stigmatized groups [56]. These guidelines speak to the acknowledged importance of building social and cultural-level processes into any intervention that hopes to address community-wide stigmatization [31,57].

Many individual-level interventions have been developed to combat stigma against certain population subgroups, including people with AIDS or mental illness. In general, these interventions have focused on educating people about the impact of stereotypes and stigmas, and on ways in which people can overcome their fears of social out-groups through sustained contact with members of those groups. Recent reviews of these studies suggest mixed results, especially in terms of sustainable effects over the long term [31,37,58]. However, one promising line of scholarship suggests the possibility of greater success with interventions that eschew traditional challenges to the “us vs. them” content of stigma and focus, instead, on enhancing the *psychological flexibility* of all parties involved. According to Masuda *et al,* psychological flexibility is “the process of engaging with private thoughts and feelings without trying to judge, evaluate, alter, fix, down-regulate or change them”. These authors, and others, suggest that a psychologically flexible person is less likely to respond to negative thoughts or feelings with maladaptive coping strategies. Interventions based on these principles attempt to undermine the psychological impact of stigmatization for all parties involved by increasing mindfulness, acceptance and perspective-taking – including the development of empathy toward self and others [59-63]. Efforts like these could be an important asset for local health officials who sense the need to develop in-depth, community-wide interventions. To be sure, it is difficult to imagine that any local health department could develop and deploy this sort of anti-stigma intervention without investing a great deal of time and energy beforehand on education and community-building. But recent studies converge on an interesting finding that could indicate a hopeful future for such interventions – namely, that people from disadvantaged social groups are not the only ones harmed by stigmatization. For example, two studies by Masuda and colleagues suggest that stigmatization directed toward other people is positively related to the *stigmatizer’s* own psychological distress [40]. And Friedman *et al* have found that perceptions of “reverse discrimination” by White people may have significant health consequences, including the hastening of inflammatory processes that could increase the risk of cardiovascular disease [39]. Armed with this knowledge, public health officials could design campaigns that increase the motivation of stigmatizing groups to curtail prejudicial statements by saying, in effect, that *“this hurts you, too!”* This notion is consistent with one of the basic tenets of stigma theory, which suggests that “undermining the distance or distinction between self and others is an important process in stigma reduction interventions” [40,59]. It is also consistent with the long-standing observation that individuals who stigmatize must be motivated to change their ways “not only because it helps those that are stigmatized, but also because it is in one’s own self-interest” [40,64].

With respect to traditionally vulnerable population groups, local practitioners should also take care to understand the particular coping resources that group members may soon be required to use; resources that may enhance their experience of self-control, optimism and social support. This is especially important when considering the case of Latino immigrants, as a prevailing backdrop of immigration-based stigma can hasten the choice of maladaptive coping strategies, including self-enforced social isolation. In addition, perceived discrimination and other psychosocial stressors may impact the body’s ability to ward off viruses, or to gain full protection from antiviral vaccines [65]. When the impact of stereotypes and stigmas is reasonably low, or when the prevailing social milieu permits open and frank discussion, Latinos and members of other disadvantaged groups can be quite resourceful in terms of protecting themselves from the flu, and in getting the support they need if they actually become ill. Here, the concept of *destino* seems important; suggesting, perhaps, that pandemic planners should direct more effort to supporting the religious and community organizations that can help local residents cultivate this resilient quality.

It is important to acknowledge the limitations of our study. Again, a small sample size means that our conclusions cannot be broadly generalized to other people and groups. And though we argue strongly for the validity of our results, we must note that they are based upon a fraction of the content available in our focus group transcripts; simply put, these groups were not designed to elicit discussion about stigmas or stereotypes. With all of this said, our study design was entirely appropriate for the task at hand. The focus group is an optimal research method for exploring people’s knowledge and experiences in their own language [66,67]. This enhances the validity of our participants’ comments, since discussions about the stigma associated with H1N1 arose organically, without heavy-handed prompts from the moderator. Focus groups are also useful for examining people’s perspectives on sensitive topics, especially when participants might otherwise be marginalized in the realm of public discussion [66-68]. The conversation that developed freely between moderators and respondents, within the safe confines of a small group, allowed for “extensive probing, follow-up questions, discussion and the observation of emotional reactions” [67]. For all of these reasons, we feel confident in our choice of research methods and in the results they produced.

## Conclusions

Our study has highlighted some of the ways in which flu pandemics can be significant sources of individual and social stress. Apart from anxiety over personal and family health, and disruptions to jobs and social relations, flu-related stress can also lead to the stigmatization of marginalized social groups. Our data show how certain elements of stigmatization – perceived and actual – were present in four Massachusetts communities shortly after the onset of the 2009 H1N1 pandemic. And apart from simply illustrating these problems, the data also suggest ways in which public health and emergency management officials can anticipate and mitigate the negative impacts of future pandemics. Armed with a thorough understanding the racial/ethnic makeup of a community – including historical patterns of stress responses and coping behaviors during emergencies – local officials can develop community-wide efforts to prepare for the possibility of stigmatization and, in some cases, to prevent it. They can work with churches and community groups to communicate about the coming threat in a way that eases social tensions and helps members of traditionally marginalized groups to draw upon their own unique resources in crafting resilient responses to pandemic outbreaks. Recent studies have also shown the importance of anti-stigma interventions that go beyond simple community-wide education about stereotypes and prejudice, and developing these interventions should be a key focus of future research. In particular, it seems important to address the underlying social and psychological factors that permit stigma to surface and persist – hence, the importance of interventions that bolster the psychological flexibility of both stigmatizers and the stigmatized.

Community resilience during pandemics and other disasters is, in the words of Shoch-Spana, “a complex process of adaptation – a collective roll with the punches – that taps into a locality’s social and material strengths” and communal stories [69]. The task of designing pandemic communication campaigns that work in harmony with these dynamics can be difficult, however, since it is not always clear that the residents who are most vulnerable – in terms of health status, socioeconomic position (SEP) and health literacy – can access, understand and act upon credible information about the risk of contracting the disease [22]. For example, people who live in high SEP communities may have access to better information about public health threats than people from low SEP communities. They may also be more likely to comprehend important health communication messages, and to take effective action based on preventive measures that are suggested. With these factors in mind, future research on stigma prevention might consider various ways in which public health practitioners can limit, or even prevent these communication inequalities when pandemics threaten their communities [22,70].

To protect the health of all Americans, public health officials must do more to combat the historical tendency to conflate the spread of infectious disease with the innate characteristics of foreigners and members of racial/ethnic minority groups. When outbreaks occur, officials must hold this tendency in check until scientists are able to convey a more accurate picture regarding the etiology of the disease in question [71]. Research on this and other communication problems during pandemic outbreaks will become increasingly important in years to come, as we strive for greater knowledge about the ways in which social and cultural forces influence the origin and propagation of disease. Armed with a better understanding of social vulnerabilities, we can develop pandemic planning policies that are more responsive to the basic human needs that arise during times of crisis [72].

### Endnotes

^a^In the present study, we use the terms *Latino* and *Hispanic* interchangeably.

^b^In labeling various coping strategies as positive or negative, we do not mean to pass judgment on any particular strategy or to imply that individual strategies are invariably good or bad.

^c^See Viswanath K, Minsky S, Ramamurthi D, Kontos EZ: **Communication under uncertainty: communication behaviors of diverse audiences during the H1N1 incidence of spring and summer 2009.** Unpublished report, Dana-Farber Cancer Institute and Harvard School of Public Health, 2009.

^d^In the following sections, we use the letter *F* to refer to female focus group participants and the letter *M* to refer to males. In passages that reproduce dialogic interactions between three or more participants, we also use numbers to distinguish between various male and female speakers.

^e^These comments come from a keynote address by David R. Williams titled *Taking Action to the Next Level: Needed Steps to Effectively Tackle Social Disparities in Health.* This address was given on September 27, 2012 at the *Leading the Way Joint Conference* in Milwaukee, WI, co-sponsored by the Medical College of Wisconsin and the University of Wisconsin School of Medicine and Public Health. For more on Williams’ assertions, see Pettigrew TF, Meertens RW: **Subtle and blatant prejudice in Western Europe.***Eur J Soc Psychol* 1995; **25:**57-75 and Williams DR, Jackson JS, Brown TN, Tones M, Forman TA, Brown K: **Traditional and Contemporary Prejudice and Urban Whites’ Support for Affirmative Action and Government Help.***Social Problems* 1999; **46:**503-527.

## Competing interests

The authors declare they have no competing interests.

## Authors’ contributions

MM designed the present study, was the primary focus group analyst, and conceptualized the manuscript. VV is Principal Investigator for the LAMPS project (see Acknowledgements); he supervised all aspects of the present study and provided scientific direction and guidance. SM is Project Director for the LAMPS study; she was responsible for study implementation and supervised data gathering throughout the focus group process. All authors reviewed MM’s analyses and contributed to successive drafts of the manuscript. All authors read and approved the final manuscript.

## Pre-publication history

The pre-publication history for this paper can be accessed here:

http://www.biomedcentral.com/1471-2458/13/1116/prepub

## Supplementary Material

Additional file 1 Focus group script. Click here for file
