# Supplementary material for: The H1N1 pandemic: media frames, stigmatization and coping
Source: BMC Public Health. 2013 Dec 3;13:1116. doi: 10.1186/1471-2458-13-1116 (PMC3907032; doi:10.1186/1471-2458-13-1116)
Supplement: Additional file 1 — Focus group script. [file 1471-2458-13-1116-S1.pdf]

FLU OUTBREAK

The following questions refer to the recent outbreak of the swine flu/H1N1.

- Where did you first hear about the swine flu/H1N1?
    - When was this?
    - How did you feel about this?

- Since then, have you actively looked for more information about the swine flu/H1N1? If so, where? (USE PROBE LIST)

- Where else have you been exposed to messages/stories/news/information about the swine flu/H1N1? (USE PROBE LIST)

- We are interested in hearing about what you heard about the swine flu/H1N1. What have you heard about:
    - The swine flu in general (what it is, how serious/dangerous it is, where it came from)
    - How the swine flu is transmitted
    - How to protect yourself from/prevent catching the swine flu
    - Swine flu vaccinations

- What sources of information have you trusted most in regards to information about the swine flu? Why?

- Did you change any of your normal behaviors or routines (ex: stop eating pork, not sending children to school) as a result of hearing about the swine flu/H1N1)?

- How have your thinking or feelings about the swine flu changed from when you first heard about it until now? Do you feel that it is more or less of a danger to you or your family?

- How do you think you or others would have reacted if this flu was called H1N1 from the beginning?
    - Do you think media coverage would have been any different?

This next set of questions refer to a future scenario:

- Suppose in the future there was a really bad outbreak of the flu in your area that put people's lives at risk. How do you think you'd find out about the outbreak? (SEE PROBE SHEET)

- What kind of information would you want before such an outbreak hit your area? How about during or after the outbreak?

- Where would you search for more information about preparing/protecting yourself from the flu? (SEE PROBE SHEET)

- How would you prefer to receive information about a flu outbreak? Why? From whom would you like to receive this information (doctor, government agency, newscaster, etc).
    - Would you consult your state or local health department at all?

- What sources of information about a flu outbreak would you trust most? Why?
  - Would you trust information provided by your state or local health department?

- Who would you want to communicate with concerning this outbreak? How would you contact them? When? Why?

- Do you have friends or family you can trust in an emergency like a flu outbreak?

- Is there any information that you would want to actively avoid hearing about concerning a flu outbreak? What? Why?

EMERGENCY PREPAREDNESS

- Experts recommend you should be prepared for emergencies. Do you know what to do in case of an emergency? Do you feel prepared?

  - How did you find out what to do?
  - Do you know where to go to obtain this kind of "preparedness" information?

- Do you or your family do anything to prepare for emergencies at this time (store flashlights, batteries, food, etc?) What do you do? Why?

- Is there anything that we haven't discussed in regards to emergency preparedness that you feel is important?

EMERGENCY COMMUNICATIONS

(IF THERE IS TIME)
We are interested in learning about is how people communicate in public health emergency situations. These include natural disasters such as hurricanes, floods or earthquakes or human-made disasters such as fires, chemical spills or accidents. We are interested in how you hear about such things, and how you communicate with other people about it. This set of questions will address this topic.

Have you ever been in any other public or community emergency situation? (natural disaster, weather emergency, chemical spill, etc?)

IF YES:

- What type of situation was it? Where/When/How many people were involved?

- Did you receive any advance warning? How? (TV, radio, etc. – SEE PROBE SHEET)
  - Did you actively look for more information on how to prepare for the situation? How? Where did you look? What types of information did you look for?

- Did you actively look for information during or after the emergency? How? Where did you look? What types of information did you look for?

- Did you have any difficulty getting information (before, during, or after the emergency)?

- Did you get information from your state or local health department at anytime during the emergency? How did you feel about the information you got from them?

- Was the information you received about the situation easy to understand/did it make sense to you? (NOTE: probe for comprehension of the information and also that it seemed reasonable to them)

- Did the information you received help you decide what to do?
  - Were you able to use the information to act, say evacuate or take action to protect yourself or your family?

- How did you use/act on any of the information that you received about the situation? How so?

- Were there any suggested actions you did not follow? Why didn't you follow them?

- How would you prefer to receive emergency communications? (SEE PROBE SHEET)

- When it comes to emergencies, a lot of rumors float around and people talk a lot. Do you have some people or communications methods/media that you trust more than others for reliable information? Who/why?

- Who did you communicate with during this time (friends/family/co-workers/emergency relief workers)? When? How?

- Is there any information any information that you actively avoided hearing, or wished you hadn't heard during this emergency? What was it? Why?
  - Are there any information sources that you actively avoided? Which? Why?

- Would you handle anything differently in an emergency situation now?

PROBE SHEET
COMMUNICATIONS METHODS/CHANNELS

TV – Which stations, specific programs – and why?

Radio – Which stations, specific programs – and why?

Internet – Any specific sites - why?

Newspaper – Which and why?

Magazines – Which and why?

Books – Which and why?

Brochures – From where? Why?

Video's/DVD – Which, from where, why?

Cell phone/PDA – how (internet/text/etc? – from whom?)

Friends/Family – how would you contact them?

Doctors/Hospitals – how would you contact them?

Employer – Who would you contact there? How would you contact them?

Schools – Who would you contact there? How would you contact them?

Government Agency – How would you contact them? Who would you contact?

Groups/Associations? (i.e. Red Cross, Medical Reserve Corp)
